# Treatment adherence and insight in schizophrenia spectrum disorders

**DOI:** 10.1192/j.eurpsy.2024.1507

**Published:** 2024-08-27

**Authors:** S. Laabidi, O. Abidi, A. Aissa, R. Hosni, U. Ouali, R. Jomli

**Affiliations:** Psychiatry A, Razi hospital, MANOUBA, Tunisia

## Abstract

**Introduction:**

Lack of adherence to antipsychotic medication in patients with schizophrenia spectrum disorders is a major risk factor for relapse and rehospitalizations which contributes to major social and economic consequences. A high proportion of patients with schizophrenia are partially or completely unaware of their mental disorder.

**Objectives:**

The aim of this study was to investigate the association between insight and medication adherence.

**Methods:**

A total number of 30 outpatients with schizophrenia spectrum disorders ,according to (DSM-V) diagnostic criteria who were attending the department of psychiatry A Razi hospital between august and September 30, 2023 were included in this study. Patients’ insight was measured by the birchwood insight scale. The degree of medication adherence was measured by using Medication Adherence Rating Scale (MARS).

**Results:**

Patients enrolled in the study had a mean (SD) age of 43.2 .There was no significant correlation between patients’ insight and patients’ ages, duration of illness and hospitalization times. In addition, there was no significant association between medication adherence and age, duration of illness, number of hospitalization or social level. Impaired insight was associated with poor antipsychotic medication adherence in patients with schizophrenia spectrum disorders .Higher insight was correlated to higher therapeutic adherence. Our results showed that the level of insight and compliance to treatment are positively correlated.

**Conclusions:**

The results of this study support the hypothesis that insight and treatment adherence are closely related. Interventions to enhance insight may be helpful in improving medication adherence.

**Disclosure of Interest:**

None Declared

